# Knockdown of circ_0003928 ameliorates high glucose-induced dysfunction of human tubular epithelial cells through the miR-506-3p/HDAC4 pathway in diabetic nephropathy

**DOI:** 10.1186/s40001-022-00679-y

**Published:** 2022-04-07

**Authors:** Qiong Liu, Yuanyuan Cui, Nan Ding, Changxue Zhou

**Affiliations:** 1 Department of Nephrology, Hebei General Hospital, Shijiazhuang, China; 2 Department of Endocrine Rheumatology and Immunology, People’s Hospital of Gaotang County, Gaotang, China; 3 Department of Clinical Laboratory, Hebei General Hospital, Shijiazhuang, China; 4 Department of Kidney Internal Medicine, Zaozhuang Municipal Hospital, No. 41 Longtou Road, Central District, Zaozhuang, 277100 China

**Keywords:** DN, circ_0003928, miR-506-3p, HDAC4

## Abstract

**Background:**

Previous data have indicated the importance of circular RNA (circRNA) in the pathogenesis of diabetic nephropathy (DN). The study is designed to investigate the effects of circ_0003928 on oxidative stress and apoptosis of high glucose (HG)-treated human tubular epithelial cells (HK-2) and the underlying mechanism.

**Methods:**

The DN cell model was established by inducing HK-2 cells using 30 mmol/L D-glucose. RNA expression of circ_0003928, miR-506-3p and histone deacetylase 4 (HDAC4) was detected by quantitative real-time polymerase chain reaction. Cell viability and proliferation were investigated by cell counting kit-8 and 5-Ethynyl-29-deoxyuridine (EdU) assays, respectively. Oxidative stress was evaluated by commercial kits. Caspase 3 activity and cell apoptotic rate were assessed by a caspase 3 activity assay and flow cytometry analysis, respectively. Protein expression was detected by Western blotting analysis. The interactions among circ_0003928, miR-506-3p and HDAC4 were identified by dual-luciferase reporter and RNA pull-down assays.

**Results:**

Circ_0003928 and HDAC4 expression were significantly upregulated, while miR-506-3p was downregulated in the serum of DN patients and HG-induced HK-2 cells. HG treatment inhibited HK-2 cell proliferation, but induced oxidative stress and cell apoptosis; however, these effects were reversed after circ_0003928 depletion. Circ_0003928 acted as a miR-506-3p sponge, and HDAC4 was identified as a target gene of miR-506-3p. Moreover, the circ_0003928/miR-506-3p/HDAC4 axis regulated HG-induced HK-2 cell dysfunction.

**Conclusion:**

Circ_0003928 acted as a sponge for miR-506-3p to regulate HG-induced oxidative stress and apoptosis of HK-2 cells through HDAC4, which suggested that circ_0003928 might be helpful in the therapy of DN.

**Supplementary Information:**

The online version contains supplementary material available at 10.1186/s40001-022-00679-y.

## Introduction

Diabetic nephropathy (DN) is the primary inducer of end-stage renal disease and is a serious complication of diabetes, featured by persistent microalbuminuria, increased blood pressure and decreased glomerular filtration rates [1]. The most significant pathological changes for DN patients are the glomerular lesions (mesangial expansion and glomerular basement membrane thickening) [2]. Besides, hyperglycemia induces oxidative stress and apoptosis of tubular epithelial cells in DN [3, 4]. At present, the multipronged drug approach by reducing proteinuria under control fails to fully prevent DN development. Thus, an in-depth investigation of the molecular mechanism related to oxidative stress and apoptosis of tubular epithelial cells in DN is required for the development of new therapeutic targets.

Circular RNA is a new member of noncoding RNAs and forms a loop through a specific event called head-to-tail splicing [5]. Owing to its wide expression in various types of cells, circRNA is attracting the attention of most investigators in revealing the pathogenesis of human diseases [6]. CircRNA governs gene expression by serving as a chelator of RNA-binding protein, a sponge for microRNA (miRNA) or a regulator of nuclear transcription [7]. Recently, research data have indicated the involvement of circRNA in renal diseases, such as renal cell carcinoma, lupus nephritis, and DN [8]. Emerging evidence indicates the function of circRNA in DN. For example, circ_010383 inhibited miR-135a to regulate renal fibrosis in DN [9]. Circ_0000064 combined with miR-143 to regulate the proliferation and fibrosis of mesangial cells in DN [10]. Circ_0003928, formed by back-splicing of TAO kinase 1 (TAOK1) gene, is one of 10 upregulated circRNAs in high glucose-induced human umbilical vein endothelial cells [11], suggesting that circ_0003928 may be involved in the pathogenesis of DN. However, the reports about the mechanism underlying circ_0003928 regulating DN development are still lacking.

miRNA, 18–25 nucleotides in length, is a noncoding single-stranded RNA that regulates gene expression post-transcriptionally [12]. miRNA regulates different biological behaviors of cells, such as cell differentiation, division, and apoptosis, thereby being involved in the pathogenesis of disease development [12]. Previous evidence has suggested the pathogenesis of DN involves some miRNAs, such as miR-342 [13], miR-337 [14] and miR-34a-5p [15]. Given that circRNA can competitively combine with miRNA through miRNA response elements to regulate the expression of the miRNA target mRNA [16], we try to assemble a circ_0003928–miRNA–mRNA regulatory axis to explain the underlying mechanism of circ_0003928 in DN development.

Thus, we hypothesized that circ_0003928 regulated DN progression through circ_0003928–miRNA–mRNA axis. The present study analyzed circ_0003928 expression in the serum of DN patients and high glucose (HG)-treated human tubular epithelial cells, investigated the effects of circ_0003928 on HG-triggered tubular epithelial cell damage, and determined a regulatory mechanism of circ_0003928 in DN development.

## Materials and methods

### Serum samples

Serum samples were collected from 41 DN patients (gender: 20 male and 21 female; age: 56.4 ± 6.2) and 29 healthy volunteers (gender: 17 male and 12 female; age: 57.3 ± 5.6) in Hebei General Hospital. The diagnosis of diabetic nephropathy was performed by two experienced renal pathologists. All serum samples were obtained by centrifugation at 3000 rpm for 10 min, and stored at -80℃ for subsequent RNA extraction. The clinical trial obtained approval from the Ethics Committee of Hebei General Hospital (approval number 2020NYH Y-010–04). All participants signed the written informed consent.

### Cell culture and DN cell model

Human tubular epithelial cells (HK-2) were purchased from Procell (Wuhan, China), and cultured in Dulbecco’s modified Eagle’s medium (DMEM; Procell) added with 10% fetal bovine serum (FBS; Procell) and 1% penicillin/streptomycin (Procell) at 37˚C in an atmosphere of 5% CO_2_. According to the reported method [17, 18], the DN cell model was established by treating HK-2 cells with 30 mmol/L D-glucose (HG; MedChemExpress, Shanghai, China), with 5 mmol/L D-glucose (NG; MedChemExpress) and 30 mmol/L Mannitol (MedChemExpress) acting as controls.

### Quantitative real-time polymerase chain reaction (qRT-PCR)

Total RNA from HK-2 cells was extracted using TsingZol (Tsingke, Shanghai, China). RNA quality was measured by detecting the A260/A280 ratio on NanoDrop-1000 apparatus. 2 μg RNA and 100 nm mRNA were reversely transcribed to cDNA using Goldenstar™ RT6 cDNA Synthesis Mix (Tsingke), gDNA remover (Tsingke) and RNasin (Tsingke). 5 μg miRNA was reversely transcribed to cDNA using miRNA synthesis reagents (TaKaRa, Dalian, China), including mRQ Buffer, mRQ Enzyme and RNase I as instructed. Then, qRT-PCR was performed to detect gene expression using T5 Fast qPCR Mix (Tsingke). In brief, 50 ng cDNA was mixed with 10 μL T5 Fast qPCR Mix (SYBR Green), 0.8 μL primers and 0.4 μL ROX Reference Dye, and reacted at 95 °C for 1 min, and 40 cycles at 95 °C for 10 s and 60 °C for 10 s on an Applied Biosystems. The 2^−∆∆Ct^ method was used to calculate gene expression with glyceraldehyde 3-phosphate dehydrogenase (GAPDH) [19] and U6 as internal controls. The primer sequences are listed in Table 1.

**Table 1:** Primers sequences used for qRT-PCR

Name		Sequences (5′–3′)
circ_0003928	Forward	CCCAACAGAAGAAAGAAC
	Reverse	CTTTGCCATCATATTGTC
HDAC4	Forward	AGCAGAGGTTGAGCGTGAG
	Reverse	GAAGTTCCCATCGTCGTAG
miR-506-3p	Forward	ACACTCATAAGGCACCCTTC
	Reverse	TCTACTCAGAAGGGGAGTAC
GAPDH	Forward	CAAATTCCATGGCACCGTCA
	Reverse	GACTCCACGACGTACTCAGC
U6	Forward	CTTCGGCAGCACATATACT
	Reverse	AAAATATGGAACGCTTCACG

### Cell transfection

The small interfering RNA against circ_0003928 (si-circ_0003928, 5′-AAGAGGATGGCCCCAGAAGTA-3′), the mimics of miR-506-3p (miR-506-3p, 5′-UAAGGCACCCUUCUGAGUAGA-3′), the inhibitors of miR-506-3p (anti-miR-506-3p, 5′-UCUACUCAGAAGGGUGCCUUA-3′), and respective controls, including siRNA negative control (si-NC), the negative control of miRNA mimics (miR-NC) and the negative control of miRNA inhibitors (anti-miR-NC) were synthesized in Ribobio Co., Ltd. (Guangzhou, China). The open reading frame of HDAC4 was amplified and then inserted into the pcDNA 3.1 vector by GenePharma Co., Ltd. (Shanghai, China) to generate the overexpression plasmid of HDAC4 (HDAC4). Then, cell transfection was performed on HK-2 cells in accordance with the manufacturer’s direction of FuGENE6 (Roche, Basel, Switzerland).

### Analysis of cell viability

To analyze cell viability, HK-2 cells treated with mannitol, normal glucose (NG), HG, plasmids, small RNA or small interfering RNA were cultured in 96-well plates for 48 h. Then, the cell counting kit-8 (CCK-8) reagent (Beyotime, Shanghai, China) was added to each well of these plates. After 4 h of incubation, these samples were analyzed using a microporous plate spectrophotometer (Bio-Rad, Hercules, CA, USA) with a wavelength of 450 nm.

### 5-Ethynyl-29-deoxyuridine (EdU) assay

After various treatments as mentioned above, HK-2 cells were cultured in 6-well plates for 48 h. Afterward, the cells were sub-cultured in the 96-well plates added with EdU-labeled DMEM at 37 °C for 2 h. Subsequent experiments were conducted according to the instruction of the EdU staining kit (Ribobio). Finally, cell proliferation was determined by analyzing EdU-positive cells using a fluorescence microscope (Olympus, Tokyo, Japan).

### Analysis of oxidative stress

The assay regarding the detection of reactive oxygen species (ROS), malondialdehyde (MDA) and superoxide dismutase (SOD) levels was performed to evaluate oxidative stress. In brief, 2 × 10^6^ HK-2 cells for each experiment were harvested, and subsequent procedures were carried out following the instruction of the cellular ROS assay kit (Abcam, Cambridge, MA, USA), lipid peroxidation MDA assay kit (Abcam), and SOD activity assay kit (Abcam). Finally, the outputs of these samples were measured by a microporous plate spectrophotometer (Bio-Rad).

### Detection of caspase 3 activity

HK-2 cells were harvested and lysed using lysis buffer (Beyotime). The cells were centrifuged at 18,000 g for 12 min, and then cell supernatant was collected. These samples were incubated with Ac-DEVD-pNA (Beyotime), and caspase 3 activity was detected using a microplate reader (Bio-Rad).

### Flow cytometry analysis

After various treatments, HK-2 cells were cultured in 12-well plates for 48 h. These cells were harvested by centrifuging and then suspended in Binding buffer (Solarbio, Beijing, China), followed by incubating with Annexin V-FITC (Solarbio) and propidium iodide (Solarbio). At last, the stained cells were analyzed using a flow cytometer (Thermo Fisher, Waltham, MA, USA).

### Western blotting

Concisely, cells and tissues were incubated with Protein Extraction Reagent (Phygene, Fuzhou, China) containing Protease Inhibitor Cocktail for 20 min on ice, and total proteins were extracted by centrifugation at 10,000*g* for 20 min. Then, 20 μg of protein were loaded onto 8%-12% bis–tris-acrylamide gels (Phygene). The proteins were wet-transferred onto nitrocellulose membranes, which were then blocked with 5% defatted dry milk. Next, the primary antibodies specific to BCL2-associated x protein (Bax; 1:1000; Thermo Fisher), cleaved caspase 3 (1:1500; Thermo Fisher), B-cell lymphoma-2 (Bcl 2; 1:50; Thermo Fisher), HDAC4 (1:1500; Thermo Fisher) and GAPDH (1:1000; Thermo Fisher) were used to incubate the membranes. After being probed with secondary antibodies (Thermo Fisher), the membranes were analyzed under a Bio-Rad image analysis system with the use of eyoECL Plus (Beyotime).

### Dual-luciferase reporter assay

Online database starbase (http://starbase.sysu.edu.cn/agoClipRNA.php?source=mRNA) was employed for the prediction of potential regulatory relationships among circ_0003928, miR-506-3p and HDAC4. Then, circ_0003928 and HDAC4 3′UTR sequences harboring the complementary sites of miR-506-3p were amplified by qRT-PCR and introduced into the pmirGLO vector to construct wild-type (WT) reporter plasmids, including circ_0003928-WT and HDAC4-3′UTR-WT. Similarly, the mutant (MUT) plasmids, including circ_0003928-MUT and HDAC4-3′UTR-MUT, were assembled. As instructed [20], cell transfection was performed on HK-2 cells with the use of the reporter plasmids, miRNA mimics, miRNA negative controls and transfection reagents. After 48 h of cell transfection, the cells were harvested for the detection of luciferase activity using a Dual-Lucy assay kit (Solarbio).

### RNA pull-down assay

The HK-2 cells cultured in 12-well plates were treated with biotin-labeled miR-506-3p mimics (Bio-miR-506-3p) and Bio-miR-NC alone. After 48 h of culture, these cells were lysed using RIP lysis buffer. After that, the supernatant was collected and incubated with streptavidin-coupled beads (Sigma, St. Louis, MO, USA). At last, circ_0003928 level in the co-precipitated RNAs was analyzed by qRT-PCR.

### Statistical analysis

All data were obtained from three independent duplicate tests and analyzed by GraphPad Prism software. Results were shown as means ± standard deviations (SD). Significant differences were compared with Mann–Whitney *U* test, Student’s *t*-tests, or analysis of variance. *P* < 0.05 indicated statistical significance.

## Results

### HG treatment induced circ_0003928 expression in HK-2 cells

**Fig. 1:**
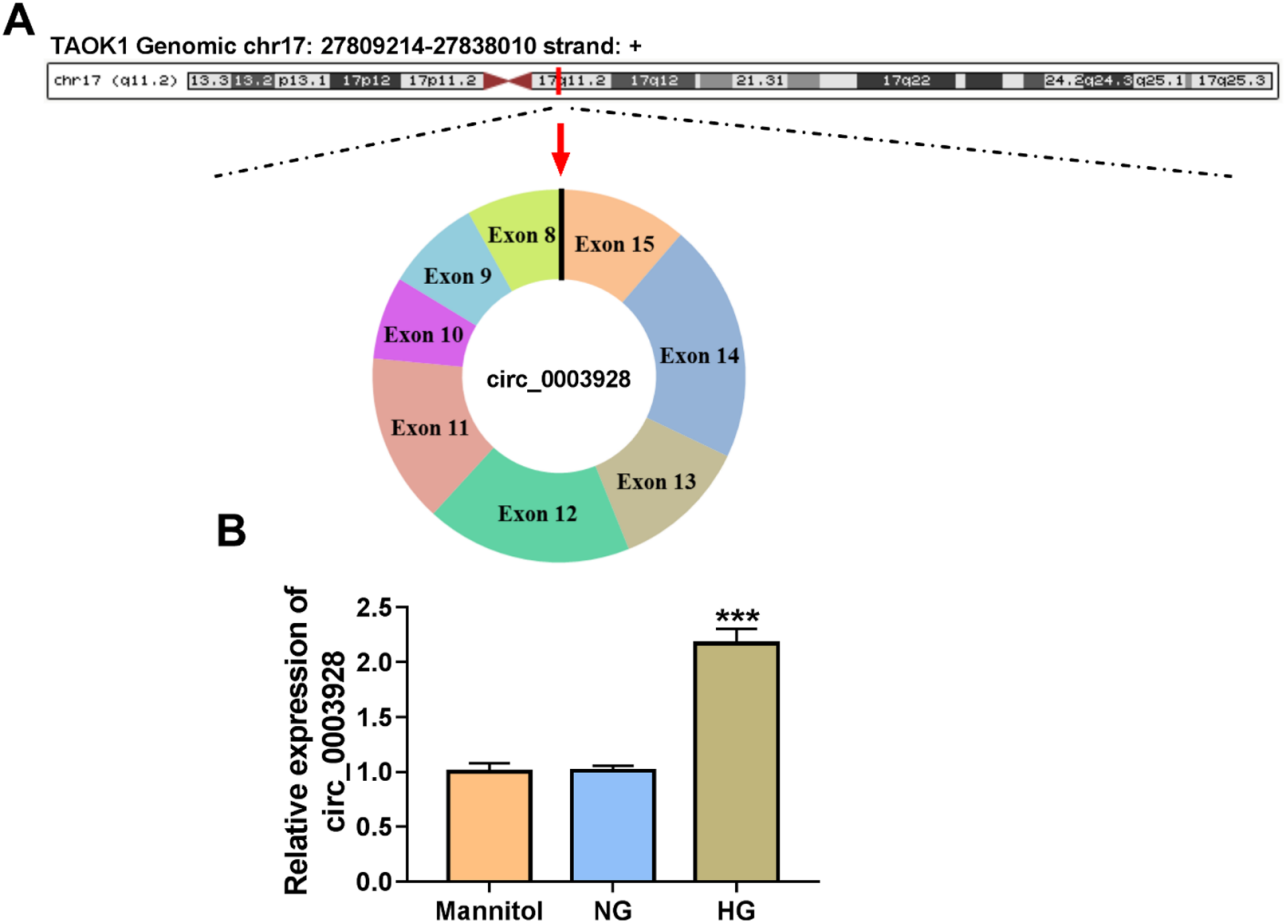
The expression of circ_0003928 in serum samples of DN patients and HG-induced HK-2 cells. **A** The schematic diagram showed the formation of circ_0003928. **B** Circ_0003928 expression was detected by qRT-PCR in the HK-2 cells induced by mannitol, NG or HG. ***P < 0.001

Circ_0003928 is located in chr17:27,809,241–27,838,010 and generated by the cyclization of exons 8–15 of the TAOK1 gene (Fig. 1A). Meanwhile, the data from qRT-PCR showed the high expression of circ_0003928 in HG-induced HK-2 cells as compared with normal glucose (NG) or mannitol-induced HK-2 cells (Fig. 1B). The above results suggest that the pathogenesis of DN may be associated with circ_0003928.

### HG treatment inhibited HK-2 cell proliferation but induced oxidative stress and cell apoptosis

**Fig. 2:**
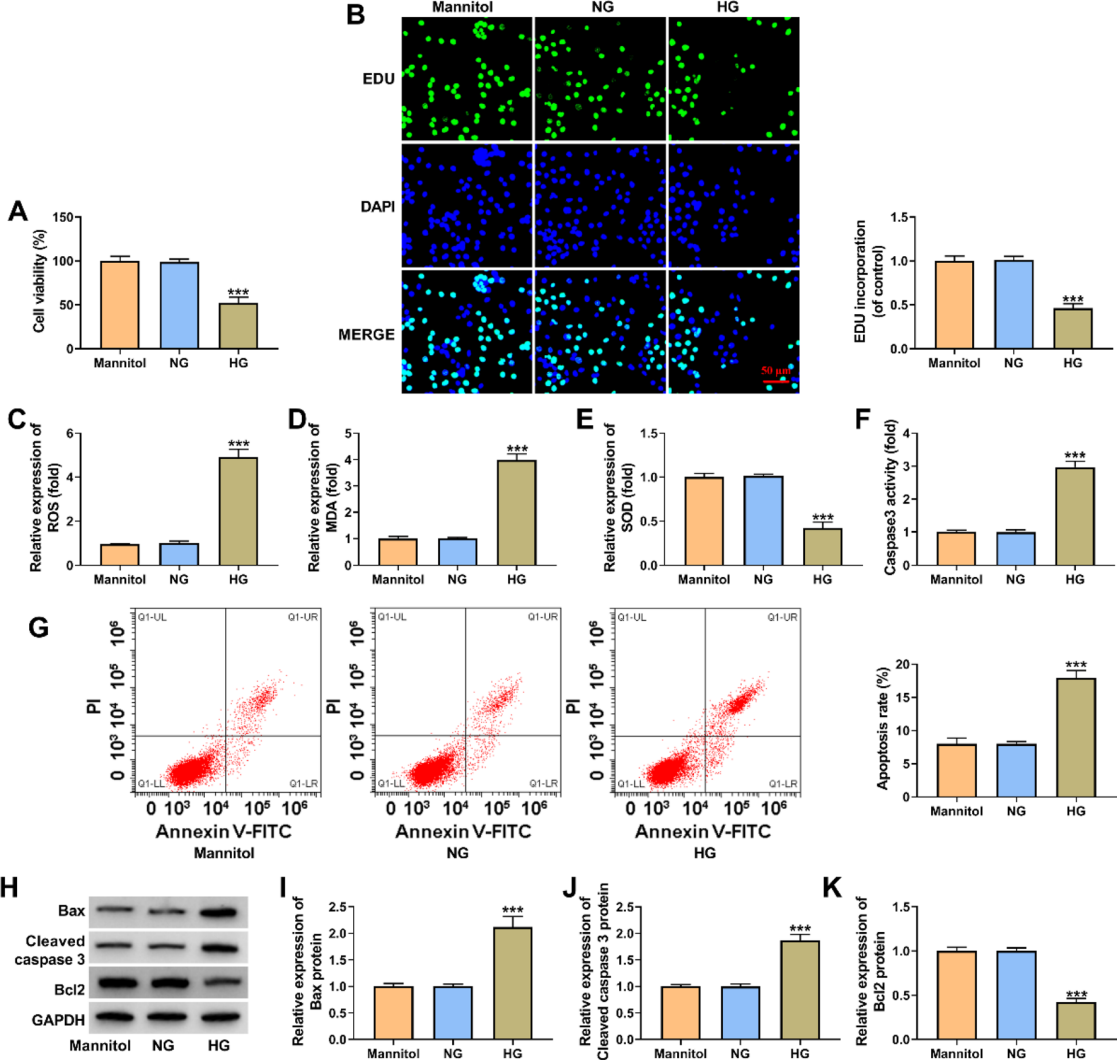
The effects of HG treatment on HK-2 cell proliferation, oxidative stress and apoptosis. A-K HK-2 cells were treated with 30 mmol/L D-glucose, 5 mmol/L D-glucose and 30 mmol/L Mannitol, respectively, and cell viability was analyzed by a CCK-8 assay (**A**), cell proliferation by an EdU assay (**B**), ROS level by a cellular ROS assay kit (**C**), MDA production by a lipid peroxidation MDA assay kit (**D**), SOD activity by a Superoxide dismutase activity assay kit (**E**), caspase 3 activity by a caspase 3 activity assay (**F**), cell apoptotic rate by flow cytometry analysis (**G**), and the protein expression of Bax, cleaved caspase 3 and Bcl 2 by Western blotting analysis (**H**–**K**). ***P < 0.001

HK-2 cells were treated with 30 mmol/L D-glucose to simulate a DN cell model in vitro, regarding 5 mmol/L D-glucose and 30 mmol/L Mannitol as controls. Subsequently, we analyzed the effects of HG treatment on HK-2 cell proliferation, oxidative stress and cell apoptosis using the model. As displayed in Fig. 2A and B, HG treatment inhibited cell viability and cell proliferation in comparison with control groups. Besides, oxidative stress was induced after HG treatment in HK-2 cells. For instance, ROS and MDA levels were increased, and SOD activity was inhibited after HG treatment (Fig. 2C–E). Comparatively, HG treatment increased caspase 3 activity and induced HK-2 cell apoptosis, accompanied by the increases of Bax and cleaved caspase 3 expression and a decrease of Bcl 2 expression (Fig. 2F–K). Collectively, the above evidence suggests the successful establishment of the DN cell model.

### Circ_0003928 depletion assuaged high glucose (HG)-induced oxidative stress and apoptosis of HK-2 cells

**Fig. 3:**
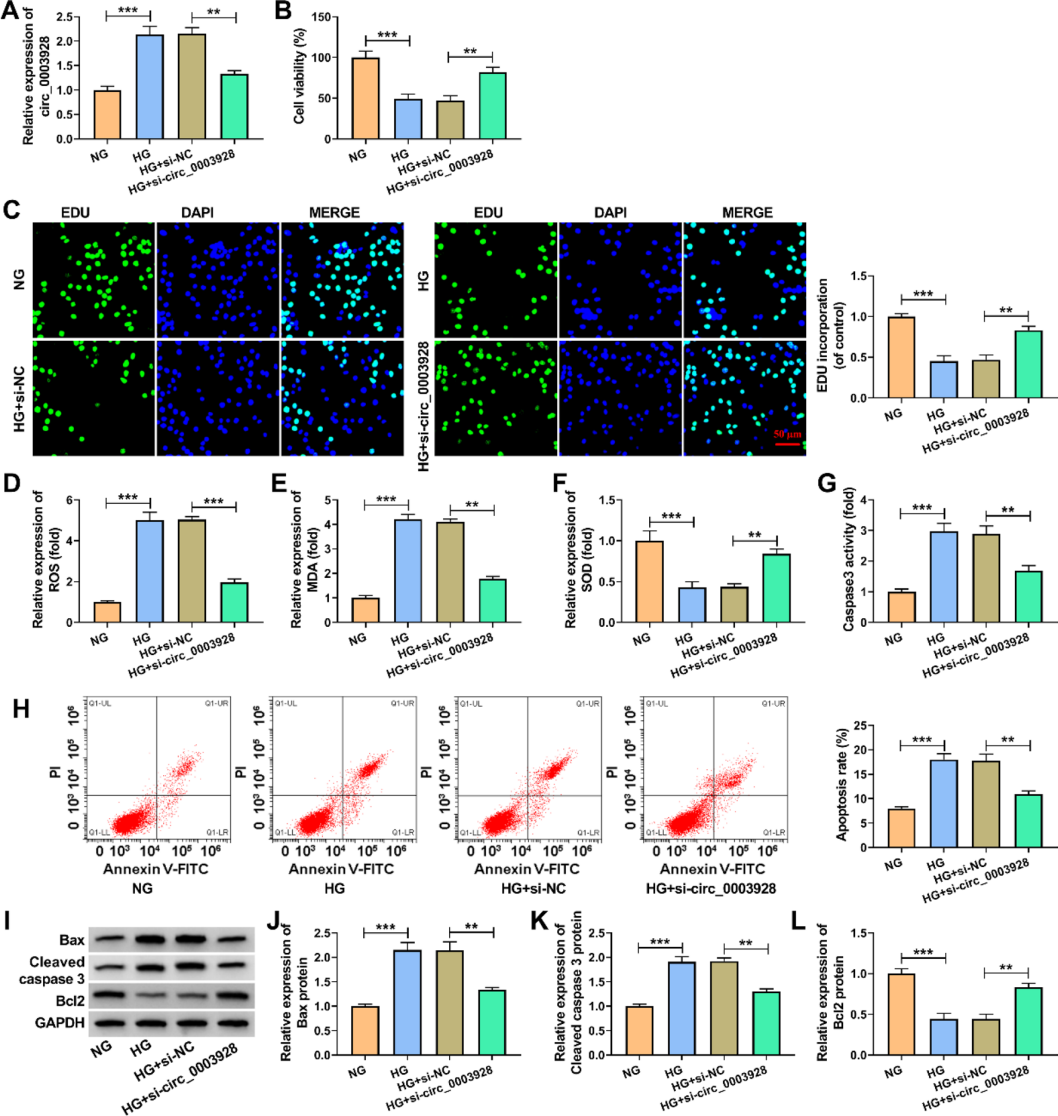
The effect of circ_0003928 depletion on HG-induced HK-2 cell damage. **A**–**L** HK-2 cells were treated with NG, HG, HG + si-NC and HG + si-circ_0003928, respectively, and circ_0003928 expression was analyzed by qRT-PCR (**A**), cell viability by a CCK-8 assay (**B**), cell proliferation by an EdU assay (**C**), ROS level by a cellular ROS assay kit (**D**), MDA production by a lipid peroxidation MDA assay kit (**E**), SOD activity by a Superoxide dismutase activity assay kit (**F**), caspase 3 activity by a caspase 3 activity assay (**G**), cell apoptotic rate by flow cytometry analysis (**H**), and the protein expression of Bax, cleaved caspase 3 and Bcl 2 by Western blotting analysis (**I**–**L**). **P < 0.01 and ***P < 0.001

We depleted circ_0003928 in HG-induced HK-2 cells to determine the consequential effects on cell proliferation, oxidative stress and cell apoptosis. The data from Fig. 3A first showed that HG-increased circ_0003928 expression was attenuated after circ_0003928 knockdown. Subsequently, HG-induced inhibition of cell viability and cell proliferation were relieved when circ_0003928 expression was decreased (Fig. 3B and C). HG treatment led to increases in OS and MDA levels and a decrease in SOD activity; however, these effects were attenuated after transfection with si-circ_0003928 (Fig. 3D–F). Comparatively, the increased caspase 3 activity and cell apoptotic rate by HG treatment were rescued after circ_0003928 depletion (Fig. 3G and H). Further, knockdown of circ_0003928 remitted HG treatment-triggered dysregulation of Bax, cleaved caspase 3 and Bcl 2 expression (Fig. 3I-L). Therefore, these findings demonstrate that circ_0003928 can regulate HG-induced oxidative stress and apoptosis of HK-2 cells.

### Circ_0003928 acted as a miR-506-3p sponge

**Fig. 4:**
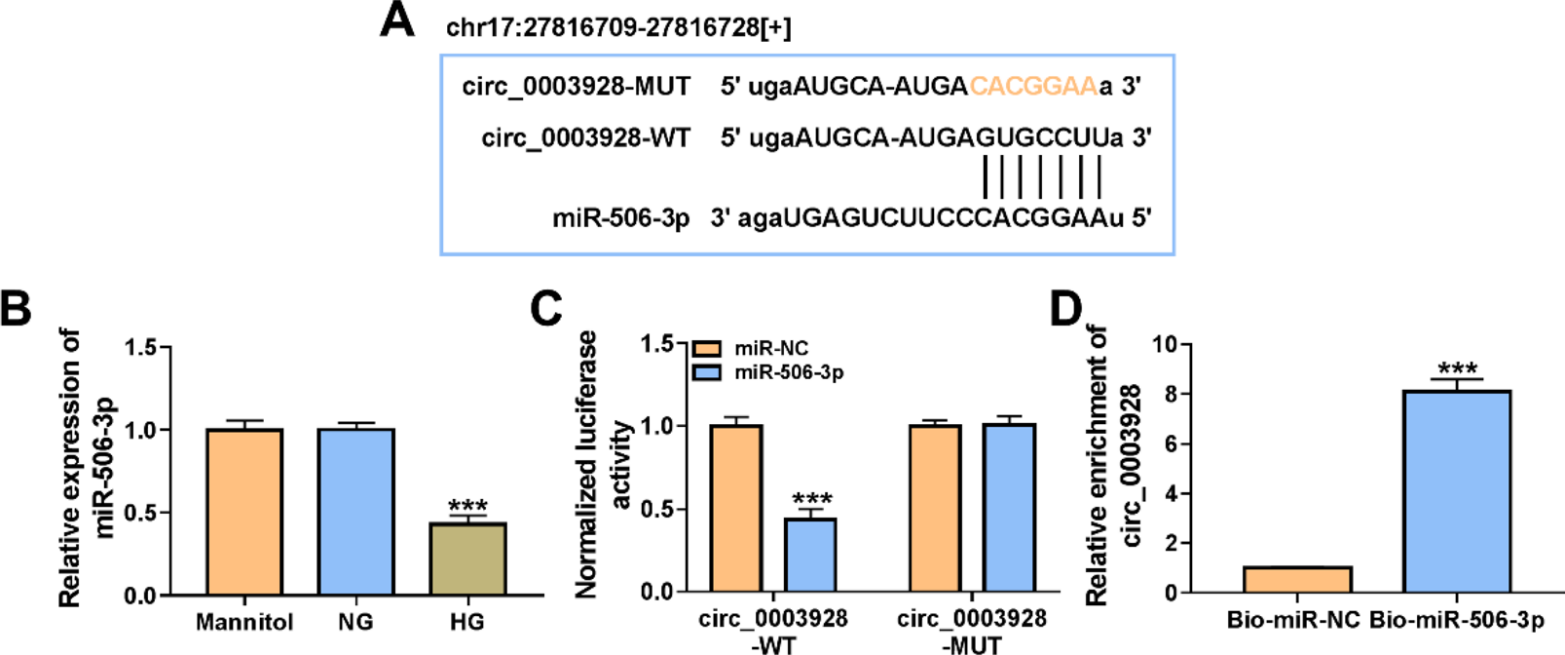
Circ_0003928 combined with miR-506-3p. **A** The schematic illustration showed the binding sites of circ_0003928 for miR-506-3p. **B** MiR-506-3p expression was detected by qRT-PCR in the HK-2 cells induced by Mannitol, NG or HG. **C** and **D** Dual-luciferase reporter and RNA pull-down assays were performed to analyze the regulatory relationship between circ_0003928 and miR-506-3p. ***P < 0.001

Starbase online database was employed to predict the possible target miRNAs of circ_0003928. In this study, we analyzed 4 miRNAs that not only contained circ_0003928-binding sites, but also inhibited DN progression, including miR-142-3p, miR-205-5p, miR-31-5p and miR-506-3p. qRT-PCR first showed that circ_0003928 depletion increased the expression of miR-142-3p and miR-506-3p, especially miR-506-3p, but it had no significant difference in miR-205-5p and miR-31-5p expression (Additional file 1: Figure S1A). Thus, miR-506-3p was chosen as a follow-up study. As presented in Fig. 4A, miR-506-3p contained the binding sites of circ_0003928, suggesting the potential interaction between circ_0003928 and miR-506-3p. Consistently, the data from the qRT-PCR analysis showed that miR-506-3p was significantly downregulated in HG-treated HK-2 cells compared with its expression in the HK-2 cells treated with mannitol or NG (Fig. 4B). Our data confirmed the interaction of circ_0003928 with miR-506-3p. For instance, miR-506-3p overexpression significantly inhibited the luciferase activity of wild-type reporter plasmid of circ_0003928 rather than that of the mutant reporter plasmid, as shown in Fig. 4C. Meanwhile, biotin-labeled miR-506-3p could greatly enrich circ_0003928 in comparison with biotin-labeled miR-NC, as analyzed by RNA pull-down assay (Fig. 4D). Further, circ_0003924 expression was negatively correlated with miR-506-3p in the blood samples of DN patients (Additional file 2: Figure S2A). Taken together, the above evidence demonstrates that circ_0003928 binds to miR-506-3p.

### Circ_0003928 regulated HG-induced oxidative stress and apoptosis of HK-2 cells through miR-506-3p

**Fig. 5:**
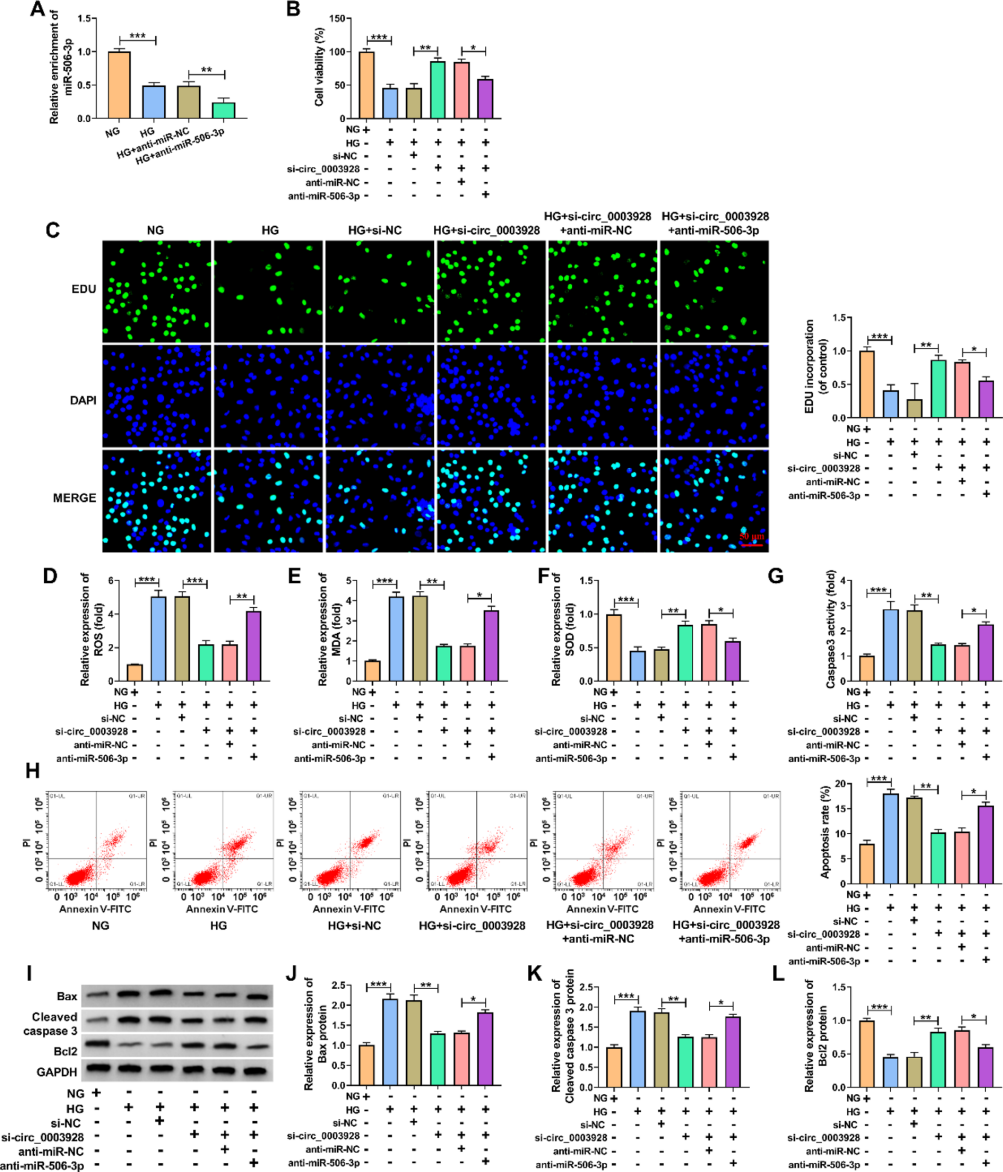
The effects between circ_0003928 and miR-506-3p on HG-induced oxidative stress and apoptosis of HK-2 cells. **A** MiR-506-3p expression was detected by qRT-PCR in the HK-2 cells treated with NG, HG, anti-miR-NC or anti-miR-506-3p. **B**–**L** HK-2 cells were treated with NG, HG, HG + si-NC, HG + si-circ_0003928, HG + si-circ_0003928 + anti-miR-NC or HG + si-circ_0003928 + anti-miR-506-3p, and cell viability was analyzed by a CCK-8 assay (**B**), cell proliferation by an EdU assay (**C**), ROS level by a cellular ROS assay kit (D), MDA production by a lipid peroxidation MDA assay kit (**E**), SOD activity by a Superoxide dismutase activity assay kit (**F**), caspase 3 activity by a caspase 3 activity assay (**G**), cell apoptotic rate by flow cytometry analysis (**H**), and the protein expression of Bax, cleaved caspase 3 and Bcl 2 by Western blotting analysis (**I**-**L**). *P < 0.05, **P < 0.01 and ***P < 0.001

Given the regulatory relationship between circ_0003928 and miR-506-3p, the study continued to analyze whether miR-506-3p participated in the regulation of circ_0003928 toward HG-induced HK-2 cell damage. To this end, we transfected si-circ_0003928, anti-miR-506-3p and matched controls into HG-induced HK-2 cells. Figure 5A first showed that HG-induced inhibition of miR-506-3p expression was enhanced after transfection with anti-miR-506-3p, showing the high efficiency of miR-506-3p knockdown. Then, circ_0003928 knockdown-induced increases of cell viability and cell proliferation were relieved after miR-506-3p knockdown under HG treatment (Fig. 5B and C). Besides, knockdown of circ_0003928 led to decreased ROS and MDA production and increased SOD activity in HG-induced HK-2 cells, but these effects were remitted when miR-506-3p expression was reduced (Fig. 5D–F). Similarly, circ_0003928 depletion decreased caspase 3 activity, cell apoptotic rate and the protein expression of Bax and cleaved caspase 3, and increased Bcl 2 expression; however, these effects were remitted after miR-506-3p depletion (Fig. 5G–L). Collectively, these findings demonstrate that the circ_0003928/miR-506-3p axis is involved in HG-induced HK-2 cell injury.

### Circ_0003928 mediated HDAC4 expression by interacting with miR-506-3p

**Fig. 6:**
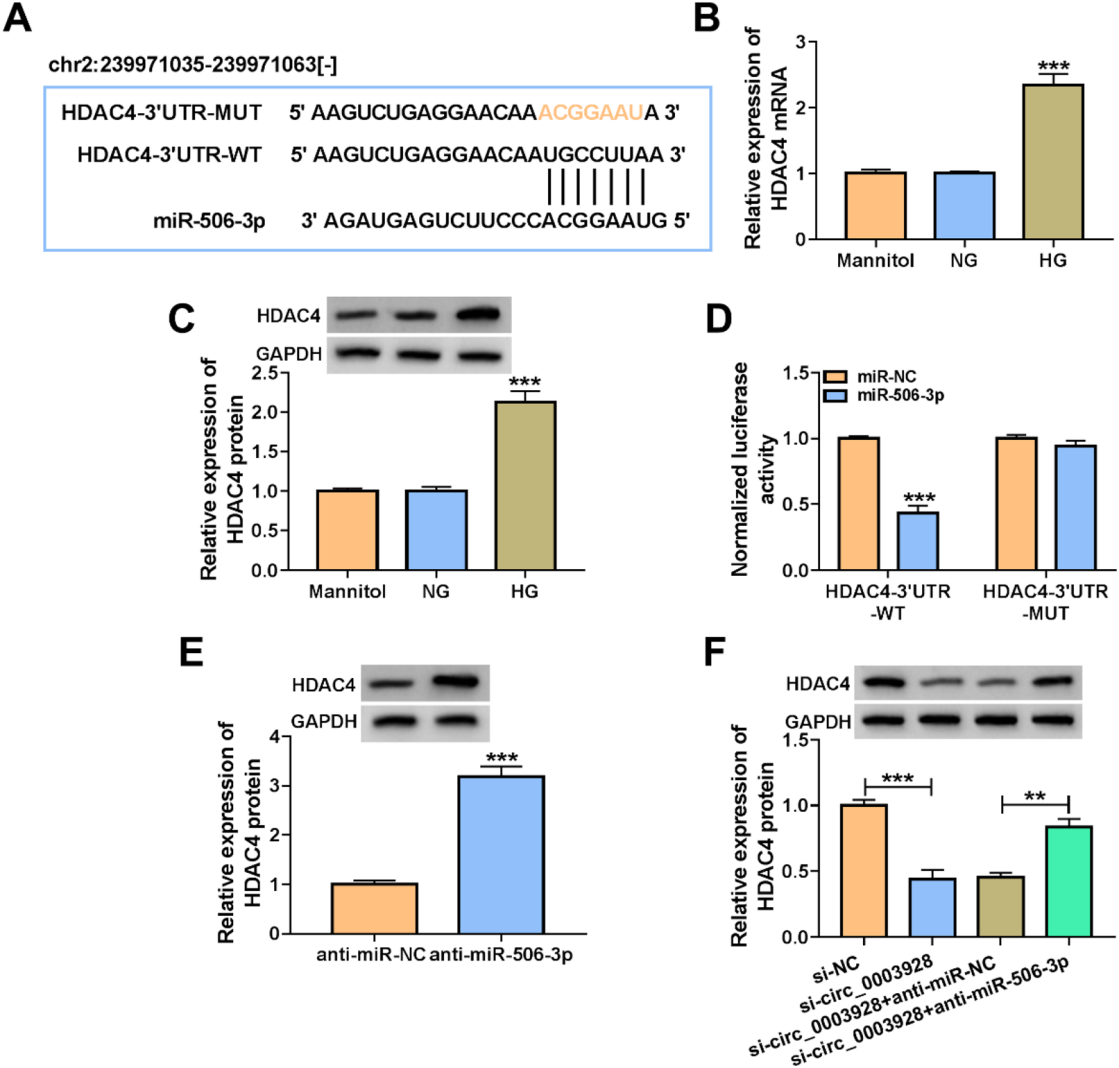
Circ_0003928 modulated HDAC4 expression through miR-506-3p. **A** The schematic illustration showed the complementary sites of miR-506-3p with HDAC4 3′UTR. **B** and **C** HDAC4 expression was detected by qRT-PCR and Western blotting in the HK-2 cells induced by Mannitol, NG or HG. **D** The dual-luciferase reporter assay was used to determine the interaction of miR-506-3p with HDAC4 3′UTR. **E** The effect of miR-506-3p depletion on HDAC4 protein expression was determined by Western blotting analysis. **F** The effects between circ_0003928 knockdown and miR-506-3p inhibitors on HDAC4 protein expression were analyzed by Western blotting analysis. **P < 0.01 and ***P < 0.001

We further identified miR-506-3p-associated mRNAs. SOX6, CDKN1A, ROCK1, NFAT5, ICAM1 and HDAC4 were chosen for subsequent studies as these mRNAs potentially bound to miR-506-3p and contributed to DN progression. By qRT-PCR analysis, we found that miR-506-3p also affected (downregulated) the expression of ROCK1 and HDAC4, especially HDAC4, among these mRNAs (Additional file 1: Figure S1B). Thus, HDAC4 was employed in the following study. HDAC4 containing the potential binding sites of miR-506-3p (Fig. 6A), as predicted by the starbase online database, was selected for the subsequent study. The study first showed a significantly high expression of HDAC4 in HG-induced HK-2 cells, as compared with control groups (Fig. 6B and C). Subsequently, we found that miR-506-3p mimics significantly reduced the luciferase activity of wild-type reporter plasmid of HDAC4 3′UTR, but that of mutant reporter plasmid had no significant difference after transfection with miR-506-3p mimics (Fig. 6D). Consistently, it was found that miR-506-3p inhibitors dramatically increased the HDAC4 expression (Fig. 6E). As revealed by Spearman correlation analysis, miR-506-3p was negatively correlated with HDAC4 expression in serum samples of DN patients (Additional file 2: Figure S2B). The above data demonstrate that HDAC4 is a target gene of miR-506-3p. Based on the above results, we analyzed the regulatory relationship among circ_0003928, miR-506-3p and HDAC4 using Western blotting. As presented in Fig. 6F, circ_0003928 depletion inhibited HDAC4 production, whereas the effect was relieved after miR-506-3p was downregulated. Taken together, all evidence suggests that circ_0003928 can regulate HDAC4 expression through miR-506-3p.

### Circ_0003928 knockdown ameliorated HG-induced HK-2 cell damage through HDAC4

**Fig. 7:**
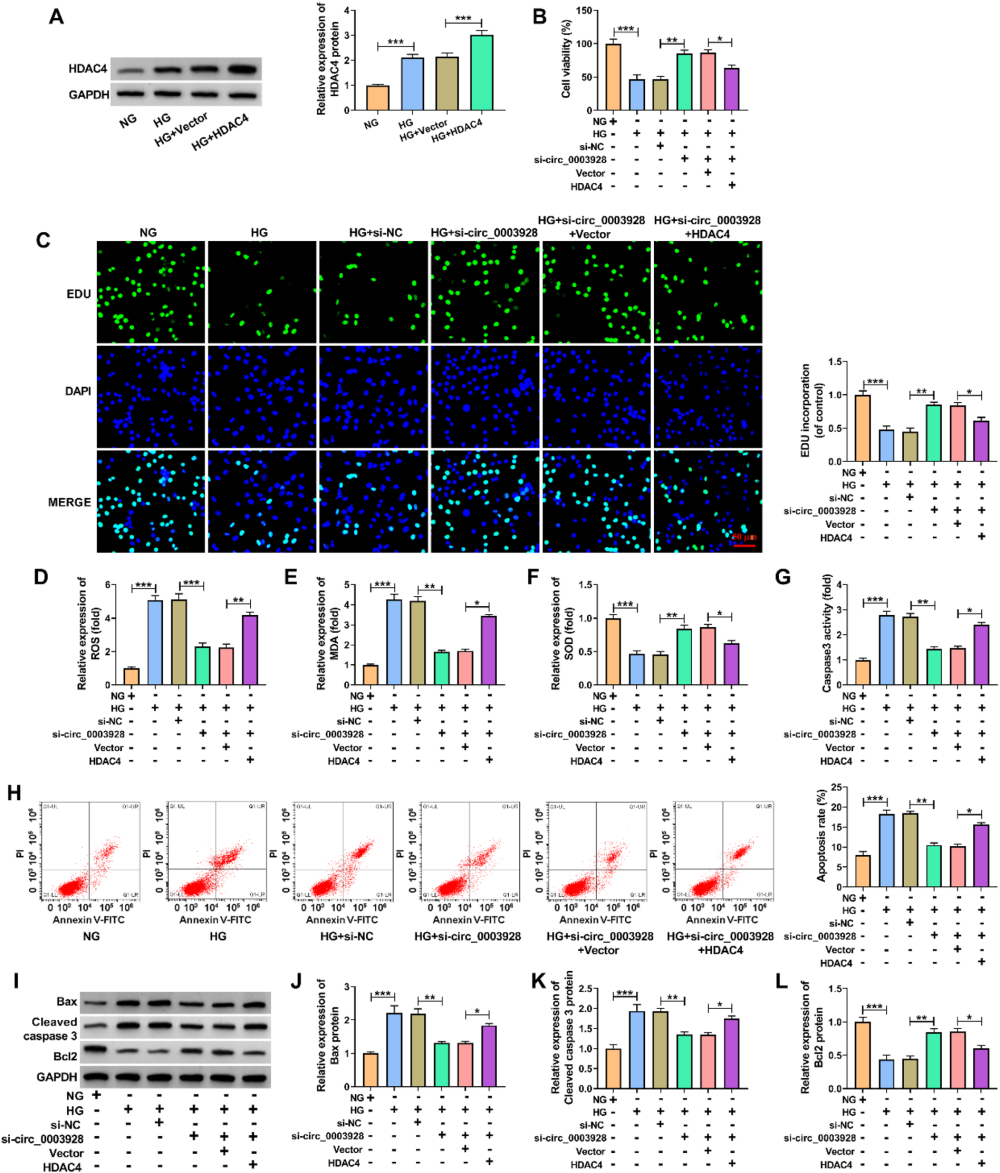
Circ_0003928 regulated HG-caused HK-2 cell disorders through HDAC4. **A** HDAC4 expression was detected by Western blotting analysis in the HK-2 cells treated with NG, HG, Vector or HDAC4. **B**–**L** HK-2 cells were treated with NG, HG, HG + si-NC, HG + si-circ_0003928, HG + si-circ_0003928 + Vector or HG + si-circ_0003928 + HDAC4, and cell viability was analyzed by a CCK-8 assay (**B**), cell proliferation by an EdU assay (**C**), ROS level by a cellular ROS assay kit (**D**), MDA production by a lipid peroxidation MDA assay kit (**E**), SOD activity by a Superoxide dismutase activity assay kit (**F**), caspase 3 activity by a caspase 3 activity assay (**G**), cell apoptotic rate by flow cytometry analysis (**H**), and the protein expression of Bax, cleaved caspase 3 and Bcl 2 by Western blotting analysis (**I**–**L**). *P < 0.05, **P < 0.01 and ***P < 0.001

Considering that circ_0003928 could regulate HDAC4 expression by associating with miR-506-3p, we further analyzed whether the inner mechanism of circ_0003928 in mediating HG-caused HK-2 cell damage involved HDAC4. As shown in Fig. 7A, HG treatment significantly promoted HDAC4 production, which was increased after HDAC4 overexpression. Subsequently, circ_0003928 knockdown promoted the viability and proliferation of HG-induced HK-2 cells, which was attenuated after HDAC4 overexpression (Fig. 7B and C). The decreased ROS and MDA production and increased SOD activity by circ_0003928 knockdown were also relieved by the enforced expression of HDAC4 in HG-treated HK-2 cells (Fig. 7D–F). Comparatively, circ_0003928 depletion inhibited caspase 3 activity, cell apoptotic rate and the protein expression of Bax and cleaved caspase 3, and promoted Bcl 2 production in HG-treated HK-2 cells; however, these effects were remitted when HDAC4 expression was increased (Fig. 7G–L). Thus, these results demonstrate that circ_0003928 regulates HG-induced cell disorders through HDAC4.

### The expression of circ_0003928, miR-506-3p and HDAC4 in the serum samples from DN patients

**Fig. 8:**
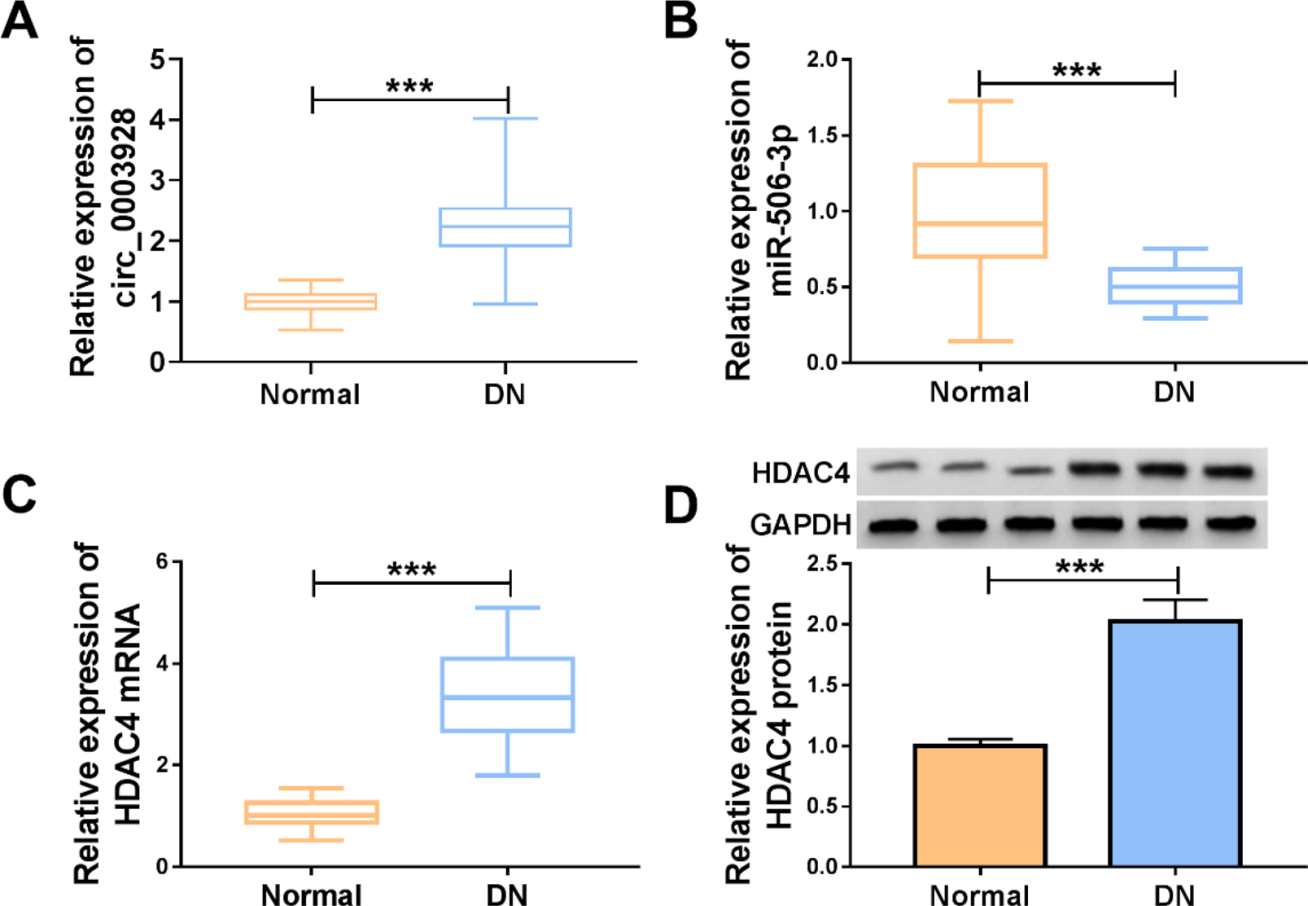
The expression of circ_0003928 (**A**), miR-506-3p (**B**) and HDAC4 (**C** and **D**) in the serum samples from DN patients and normal volunteers (N = 29) was analyzed by qRT-PCR or Western blotting. ***P < 0.001

The study further detected circ_0003928, miR-506-3p and HDAC4 expression in the serum samples from DN patients and healthy volunteers. As shown in Fig. 8A, circ_0003928 was significantly upregulated in the serum samples of DN patients compared with healthy controls. Subsequently, we observed a lower level of miR-506-3p and a high HDAC4 expression in the serum samples of DN patients compared with control groups (Fig. 8B–D).

## Discussion

Recently, increasing evidence suggests that circRNAs are involved in the mechanism of DN progression [21]. In the present work, we analyzed the function of circ_0003928 in HG-induced human tubular epithelial cell dysfunction and disclosed the underlying mechanism. As a result, we found that circ_0003928 participated in regulating proliferation, oxidative stress and apoptosis of HG-treated human tubular epithelial cells through the miR-506-3p/HDAC4 pathway.

TAOK1, a parental gene of circ_0003928, is a member of human TAO kinase orthologs (TAOKs) that participate in multiple signaling pathways, such as the JNK/SAPK cascade, the p38 MAPK pathway and the Hippo signaling pathway [22]. Through these interactions, TAOKs regulate cytoskeleton, inflammatory response, oxidative stress and apoptosis and other cellular pathways [3]. Oxidative stress is regarded as an important factor associated with hyperglycemia [3]. Hyperglycemia accelerates apoptosis of different cells, such as tubular epithelial cells and endothelial cells, through activation of some signaling pathways, leading to renal injury in DN [23, 24]. In this work, we analyzed the role of circ_0003928 in HG-caused oxidative stress and apoptosis of human tubular epithelial cells. Herein, we found a high expression of circ_0003928 in the serum of DN patients and HG-treated human tubular epithelial cells, and the promoting effect of circ_0003928 on HK-2 cell apoptosis, which were consistent with the published data [18]. In addition, the work provided evidence that circ_0003928 silencing attenuated HG-induced proliferation inhibition and oxidative stress promotion.

miRNA is a short noncoding RNA that has attracted much attention as an important regulator of DN progression [25]. Considerable studies suggest that circRNA contains the complementary sites of miRNA, and combines with miRNA [26]. Herein, we found that miR-506-3p combined with circ_0003928. As reported early, miR-506-3p regulates the progression of cancer [27], atherosclerosis [28], bronchial asthma [29] and cardiovascular disease [30]. In particular, miR-506-3p combined with long noncoding RNA KCNQ1OT1 (lncRNA KCNQ1OT1) to regulate HK-2 cell oxidative stress and pyroptosis [17]. The present study suggested that miR-506-3p promoted HK-2 cell proliferation. Besides, miR-506-3p inhibited HK-2 cell oxidative stress and apoptosis. Importantly, we provided evidence that circ_0003928 regulated HG-induced HK-2 cell dysfunction through miR-506-3p.

The HDAC family is a class of enzymes able to modulate acetylation activities of histone acetyltransferases, playing important parts in mediating gene transcription [31]. There are data suggesting the involvement of the HDAC family in DN progression through various molecular pathways [32]. Among several HDACs, HDAC4 is highly expressed in DN and is a major contributor to podocytes’ injury [33]. Mechanistically, HDAC4 contributes to podocyte dysfunction through several mechanisms, such as interacting with miR-29a [34] and upregulating calcineurin [35]. In the present investigation, HDAC4 was associated with miR-506-3p. HDAC4 was highly expressed in DN patients and HG-induced HK-2 cell samples. Besides, HDAC4 overexpression attenuated circ_0003928 depletion-mediated effects in HG-induced HK-2 cells. Further, circ_0003928 negatively regulated miR-506-3p to promote HDAC4 production.

## Conclusion

Taken together, the present study demonstrates that circ_0003928 is a crucial regulator of the signal transduction pathway related to HG-induced HK-2 cell dysfunction in DN. HG-induced HK-2 cell injury involved the upregulation of circ_0003928. The increased expression of circ_0003928 induced HDAC4 production by sponging miR-506-3p, further inhibiting cell proliferation and promoting oxidative stress and cell apoptosis (Additional file 3: Figure S3). Interfering the circ_0003928/miR-506-3p/HDAC4 axis may be an effective method for the therapy of DN. Besides, tubular epithelial cell-specific circ_0003928 knockdown diabetic db/db mice will be an ideal model for analyzing the role of circ_0003928 in DN in vivo. Thus, our findings will be further testified in subsequent investigations using the genetic model. Previous studies have shown that the Nrf2/heme oxygenase-1 (HO-1) pathway regulates diabete-caused kidney damage [36], and that miR-505-3p aggravates oxidative stress injury through the Nrf2/HO-1 signaling pathway [37]. Thus, whether circ_0003928-mediated HK-2 cell dysfunction involves the pathway will be explored in the future.

## Supplementary Information

**Additional file 1: Figure S1.** Circ_0003928-associated miRNAs and miR-506-3p-associated mRNAs were analyzed by qRT-PCR in HK-2 cells. *P < 0.05, **P < 0.01 and ***P < 0.001.

**Additional file 2: Figure S2.** Spearman correlation analysis was performed to determine the correlation between miR-506-3p and circ_0003928 (A) or HDAC4 (B) in the serum samples of DN patients.

**Additional file 3: Figure S3.** The schematic illustration showed the mechanism by which circ_0003928 regulated HG-induced HK-2 cell dysfunction. HG-induced HK-2 cell injury involved the upregulation of circ_0003928. The increased expression of circ_0003928 induced HDAC4 production by sponging miR-506-3p, further inhibiting cell proliferation and promoting oxidative stress and cell apoptosis.

## Data Availability

The analyzed data sets generated during the study are available from the corresponding author on reasonable request.
