# Supplementary figures and images for: Knockdown of circ_0003928 ameliorates high glucose-induced dysfunction of human tubular epithelial cells through the miR-506-3p/HDAC4 pathway in diabetic nephropathy
Source: Eur J Med Res. 2022 Apr 7;27:55. doi: 10.1186/s40001-022-00679-y (PMC8991937; doi:10.1186/s40001-022-00679-y)

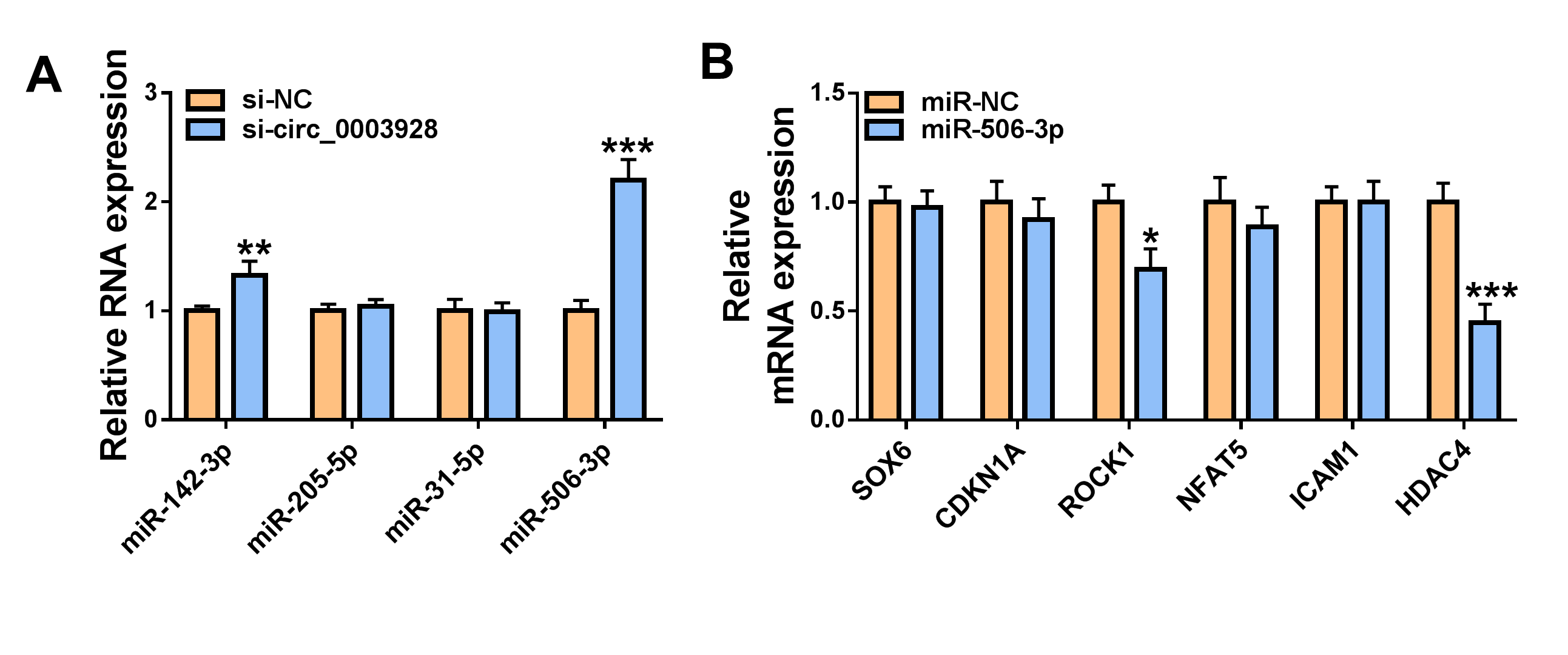

Supplement: Supplementary file 1 — Additional file 1: Figure S1. Circ_0003928-associated miRNAs and miR-506-3p-associated mRNAs were analyzed by qRT-PCR in HK-2 cells. *P < 0.05, **P < 0.01 and ***P < 0.001. [file 40001_2022_679_MOESM1_ESM.tif]

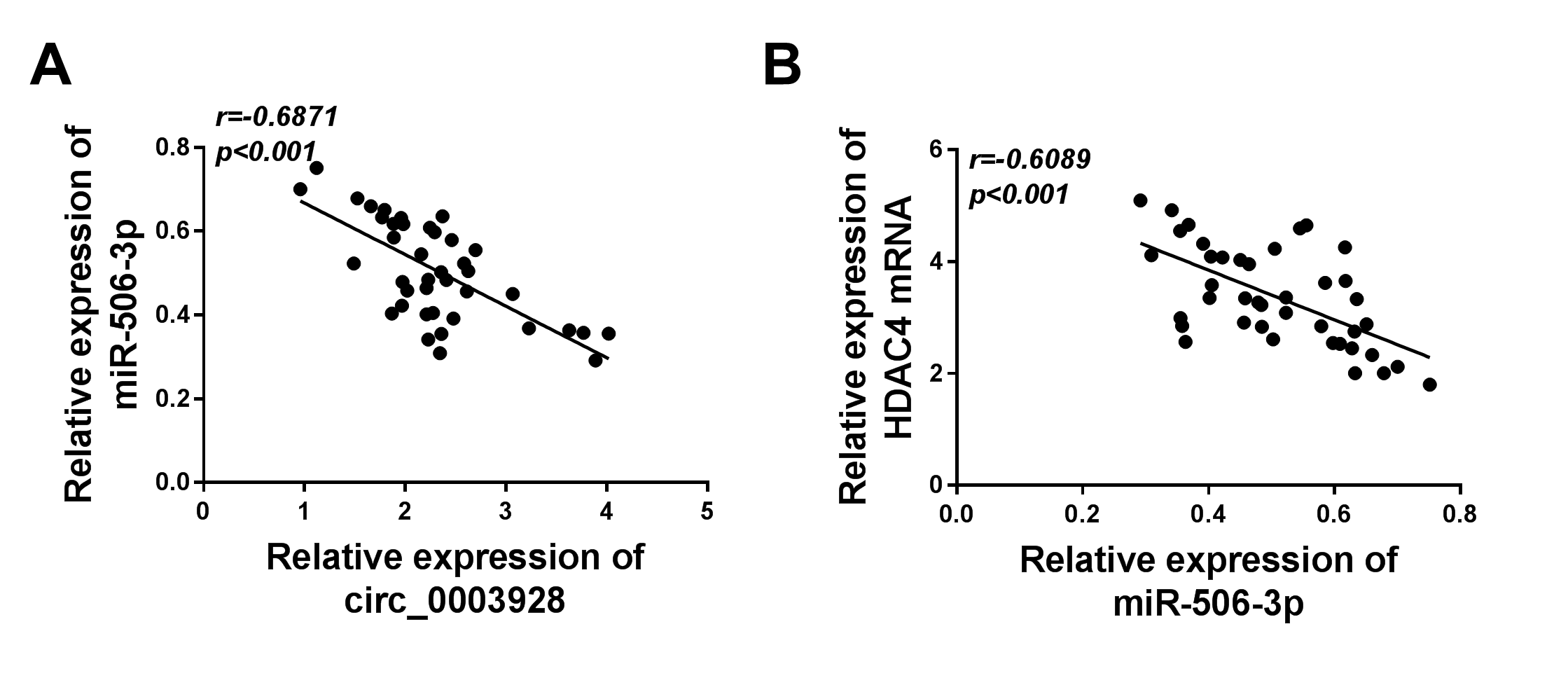

Supplement: Supplementary file 2 — Additional file 2: Figure S2. Spearman correlation analysis was performed to determine the correlation between miR-506-3p and circ_0003928 (A) or HDAC4 (B) in the serum samples of DN patients. [file 40001_2022_679_MOESM2_ESM.tif]

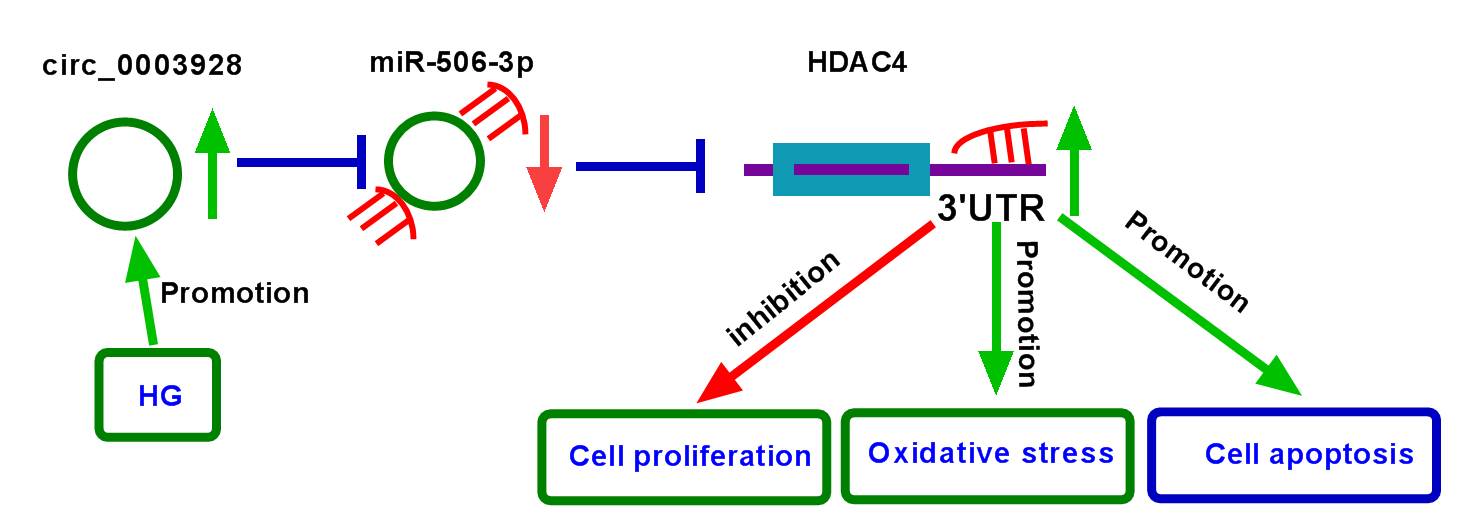

Supplement: Supplementary file 3 — Additional file 3: Figure S3. The schematic illustration showed the mechanism by which circ_0003928 regulated HG-induced HK-2 cell dysfunction. HG-induced HK-2 cell injury involved the upregulation of circ_0003928. The increased expression of circ_0003928 induced HDAC4 production by sponging miR-506-3p, further inhibiting cell proliferation and promoting oxidative stress and cell apoptosis. [file 40001_2022_679_MOESM3_ESM.tif]
